# Medical Notes and Comments

**Published:** 1878-05

**Authors:** Charles A. Wall


### MEDICAL NOTES AND COMMENTS—BY CHARLES A. WALL, B. S

In our last we gave Prof. YANDELL credit for writing upon 'Malaria and Stuma.' It was not our object to thus misrepresent the talented author, and will ask all readers of that article to substitute 'Struma' for 'Stuma.'——ERICHSEN is out with an eighth and enlarged edition of his exhaustive work. He has been standard authority on Surgery for thirty years, and is more worthy of confidence than ever. Many parts of the new Edition have been entirely rewritten, and all subjects are brought down to correspond with the latest ideas.——JOHN WYETH & BRO. send us an elegant price-list of Pharmaceutical preparations, compressed powders, and medical fluid extracts. It is supplied upon application, and will be found useful in ordering medicines.——Dr. A. B. LYONS read an exhaustive paper before the Detroit Academy of Medicine, and reprints it from *Detroit Lancet*, Feb. and March, 1878, upon 'Medical Plants Indigenous in Michigan.'—'Our Parks to be or not to be,' by Edward Squibb, M. D. is a strong protest against their destruction. He says: "To save our Parks will save hundreds of children and improve thousands. To not improve these Parks is to abandon them to the beast of prey, which is devouring American civilization."——The average weekly cost of keeping the paupers of the Eighth Judicial District, is, we learn from the report of Hon. Wm. P. Letchworth, about $1.31 each.——'Influence of School-life on Eye-sight,' by A. W. CALHOUN, M. D., reprint from *Atlanta Medical and Surgical Journal*, is an article asking for hygienic school-houses, well lighted, and text-books printed in clean type.——'What am I,' by J. M. BODINE, M. D., leaves the question unsolved, but contains many instructive thoughts.——Three hundred and ninety-eight operations were performed at the Ophthalmic and Aural Institute, 46 E. 12th St. N. Y. City during 1877, according to the Eighth Annual Report.——Total cost per week exclusive of buildings and extraordinary repairs for each patient at the Buffalo General Hospital for the past year, was $6.10; Annual Report for 1877,——The daily number of Free Beds in the Massachusetts General Hospital during 1877 (Sixty-fourth Annual Report,) was one hundred and forty. The total number admitted since 1821, has been over forty-eight thousand.——Louisiana has, at last, formed a State Medical Association. Its first meeting began Jan. 14th, 1878, and lasted three days. A Constitution and By-Laws were provisionally adopted. Dr. Egan was elected chairman, and papers were appointed to be read at the next meeting.——Dr. H. KNAPP reprints from the *Archives of Ophthalmology and Otology*, vol. VI, No. I-II, his Report and Remarks on the fourth and fifth hundred of Cataract Ex tractions, according to Von Graeffe's method. The results were good in 164 cases or 82 per cent; his failures were 23 or 11.5 per cent—— Dr. LEE, in a paper upon. 'Suspension as a Means of Treating Spinal Distortions, read before the American Medical Association, fully demonstrates the use, and in many cases necessity of removing the superincumbent weight of the head and body in Potts Disease, and the benefit derived from exercise in lateral curvature.

